# Mental Simulation to Promote Exercise Intentions and Behaviors

**DOI:** 10.3389/fpsyg.2021.589622

**Published:** 2021-11-16

**Authors:** Weitan Zhong, Guoli Zhang

**Affiliations:** School of Psychology, Beijing Sport University, Beijing, China

**Keywords:** mental simulation, process simulation, outcome simulation, exercise, emotion

## Abstract

Mental simulation, which employs specific patterns of imagery, can increase the intention to exercise as well as actual engagement in exercise. The present studies explored the effects of mental simulation on the intention to engage in exercise while regulating emotions. The first study confirmed that mental simulation did promote intentions of participants. The second found that video-primed mental simulation was a more effective method of exercise intention promotion than semantic-primed and image-primed mental simulation. In the third study, it was found that combining process-based and outcome-based mental simulations increased exercise intentions. Positive emotions mediated imagery ability and intention to exercise. The final study found that the mental simulation interventions most effective for exercise adherence were those that balanced the valence of process and outcome components in such a way that a challenging process results in a positive outcome, or a smooth process results in a negative outcome. Each of these results has practical implications for exercise interventions that will be discussed.

## Introduction

Mental simulation helps individuals relieve stress, organize their daily activities more effectively, and achieve their personal goals (Ignacio et al., 2017). In fact, it was found that inducing an imagined experience provides an emotional experience similar to what is provided by an actual positive exercise experience (Stevens et al., 2019); additionally, previous work has revealed that a positive exercise experience can increase exercise intention and activity (Caplandies et al., 2018). Taylor et al. (1998) studied a group of individuals seeking to lose weight and showed that the most effective method involves simulating one’s successful achievement of a goal through a specific weight-loss process. Positive outcome simulation allows individuals to achieve a positive emotional experience, and process simulation enables them to imagine every aspect of achieving a goal in detail (Hagger, 2017), improving their problem-solving capacities, behavioral control, and capacities to achieve a goal. It, therefore, appears that positive emotion, relatively to either an outcome or a process, is central to the effect mechanism of mental simulation on intentions and achievement.

Most previous studies have used semantic priming (Wilson-Mendenhall et al., 2018); some have used image priming (de Koning et al., 2016; Mannaert et al., 2017), but a few have applied video priming. Video-primed imagery training was effectively used to improve skills of gymnasts (Battaglia et al., 2014), but it remains to be determined whether videos are more effective than other priming methods. Studies on different forms of mental simulation (Hammell and Chan, 2016; Marszał-Wiśniewska and Jarczewskagerc, 2016) have found that the effects of prefactual thinking simulation on the performance of physical tasks are superior to the effects of counterfactual thinking. However, it is not known how the different types and content of process simulation differentially affect intentions of people. The present studies examined the different types and content of mental simulation and investigates the efficacy of mental simulation in promoting exercise intention and behavior. More specifically, these studies explore whether video-primed mental simulation is the most effective of the three priming methods and whether positive emotion is a significant mediator of the relationship between mental simulation and exercise intentions.

## Mental Simulation of Exercise

Exercise is gradual, persistent physical activity that is planned, formulated, and repeated (Caspersen et al., 1985, p. 128). It can improve or maintain one’s physical fitness, improving cardiopulmonary functioning, muscle strength, body composition, and flexibility (Puetz et al., 2006). It also has mental health benefits, such as elevating self-regulation and self-esteem and decreasing depression and anxiety (Fontaine, 2000).

Interventions that help individuals initiate and maintain regular exercise have some effect but lack long-term impact, since studies have found a gap between intentions and behavior (Orbell and Sheeran, 1998; Rhodes and Bruijn, 2013; Sniehotta et al., 2014; Sheeran and Webb, 2016). Indeed, studies have shown that individuals who are classified as “abstainers” (Orbell and Sheeran, 1998) or as having “unsuccessful intentions” (Rhodes and Bruijn, 2013) may have strong intentions to engage in healthful behaviors but may encounter many difficulties in translating those intentions into actions, ultimately leading to abandonment. Mental simulation, involving the realization of personal imagined goals (e.g., imagining the emotions, situations, and thoughts experienced when a goal is achieved), is a strategy that individuals can use to visualize their path toward certain goals and strengthen their behavioral intentions by improving their individual tendencies and readiness for action (Pham and Taylor, 1999).

Marszał-Wiśniewska and Jarczewskagerc (2016) conducted two experiments to verify the effects of various mental simulations on the effectiveness and sustainability of the weight-loss process. The two studies demonstrate the self-regulatory benefits of mental simulation in pursuing challenging long-term personal goals. The findings show that even mental simulations of very difficult tasks can promote successful goal achievement. It shows that it is not necessary to negate the effects of outcome simulations-they must be accompanied by simulations of favorable (for negative outcomes) or unfavorable (for positive outcomes) processes. Another important finding of this study was that mental simulation can affect effectiveness and improve behavioral persistence. Mental simulations can be particularly useful when setting goals, especially novel or complex goals that require elaboration. However, once a goal achievement strategy is in place and the best ways to succeed have been established, a more important task may be to form and maintain implementation intentions (Gollwitzer, 1999; Hagger et al., 2016). Due to the influence of time, even if a goal has been set and the required actions have been simulated in advance, the presimulated behavior is often replaced with a practical solution as the agent approaches the goal.

## Mental Simulation and Emotion

Grounded cognition (Barsalou, 2008) and embodied cognition hold that human cognition is inseparable from the body, the environment, and society. They organically combine rather than separate. Personality, worldview, belief, and temperament differences inspire different mental simulations. Even when the same event is simulated, individuals may experience different emotional experiences and encounter a different state. Since mental simulation can shape an emotional experience of an individual, it will also affect the physical state the individual, such as his or her heart and breathing rate, blood pressure, hormone secretion, and electrophysiology. Thus, exercise mental simulation regulates emotions of exercise experience.

The emotional functions of mental simulation can be realized through two means: emotional comparison and emotional assimilation. Emotional comparison involves an individual imagining a fictitious scene or event and then comparing this visualization to actual conditions. During emotional assimilation, only emotional experiences or adaptations to an imagined scene or event are considered. Szpunar and Schacter (2013) showed that repeated mental simulations can enhance one’s rationality in addressing future emotional interpersonal events and that this rationality is related to the difficulty, specificities, and impacts of simulated events.

The mental image involved in mental simulation is not just a figure or schema but, rather, is embodied in a human-like system derived from rich perception. Negative emotions in particular stimulate counterfactual (Johnson-Laird and Oatley, 2000) and more detail-oriented thinking (Schwarz and Clore, 2007). Rivkin and Taylor (1999) explored the role of mental simulation in responses to controllable stressful events and found that structured mental simulations of continuous, controllable stressful events can improve both emotional experiences associated with events and proactive approaches to problem-solving. The authors also found that, to regulate emotions, simulating uncontrollable events is more effective, while, to solve problems, simulating controllable events is more effective.

## Mental Simulation: Process- and Outcome-Based Simulation

Taylor and Schneider (1989) formally proposed the concept of “mental simulation,” which involves imagining the functions or processes of certain events or series of events through counterfactual thinking. In addition, mental simulation involves imagining future activities through prefactual thinking. As outcome- and process-based simulations implicitly refer to the future, the current program of studies focuses on prefactual thinking. However, this research does not rule out the possibility of individuals drawing on counterfactual thinking simulations of previous experiences.

Mental embodied practices are process simulations emphasized in mental simulation (Pham and Taylor, 1999). Those who engage in process simulation are more likely to monitor their own behaviors and improve their planning and rational analysis capabilities. They are also more inclined to imagine the actions to take or avoid in pursuit of their goals, ultimately leading them toward goal-oriented action (e.g., simulating physical sensations, emotions, or environmental settings). Studies have indicated that mental simulations can effectively promote behavioral participation and goal achievement in many areas, including health (Pham and Taylor, 1999; Vasquez and Buehler, 2007; Hagger et al., 2012).

Outcome simulation involves the mental simulation of targeted results (Pham and Taylor, 1999). Positive outcome simulation involves goal setting and imagining achieving a goal to stimulate motivation of an individual to take practical action while strengthening his or her sense of self-efficacy. Negative outcome mental simulation involves imagining not achieving a goal. Outcome simulations often use the affirmative phrase “I can do it” to keep individuals working hard to realize their potential (Ruvolo and Markus, 1992).

## Research Program

The present research program ultimately aimed to divide process simulations into *smooth* and challenging simulations. Challenging process simulation requires people to simulate a difficult process in which events cannot be fully guided by goals. Moreover, we wondered whether a combination of process and outcome simulation could achieve a wide range of effects on people. Few studies (Lee et al., 2018; Kang et al., 2019; Svingen et al., 2020) have conducted multiple continuous interventions in humans. Mental simulation as a one-time intervention (Koka and Hagger, 2016) has the immediate effect of improving exercise maintenance of an individual; however, the cited study did not determine whether it was more effective than a one-time intervention at influencing continuous behaviors.

“Study 1” tested whether mental simulation could promote the intention to engage in exercise. “Study 2” explored the effects of mental simulation of different priming methods (semantics, images, and videos) on the intention to participate in exercise. “Study 3” mainly explored the different types of mental simulation (outcome simulation: positive, negative; process simulation: smooth, challenging) and which method is most effective in promoting the intention to engage in exercise. “Study 4” mainly explored the effect of the type and times of mental simulations on exercise behavior in a week.

## Study 1

Preplanning and problem-solving through prefactual mental simulations can help individuals adapt to future events (Arnold et al., 2016) *via* prospective memory, decision-making, and emotional regulation. A one-factor (prime vs. control) between-subjects experimental design was applied in “Study 1” to determine whether mental simulation has a priming effect on the intention to engage in exercise. The independent variable was mental simulation, and the dependent variable was exercise intention.

### Methods

#### Participants

We recruited 72 ordinary college students (59 female participants, *M*_age_ = 20.82, *SD* = 2.55) from a university in Beijing *via* advertisements, and some students participated for extra course credit. This sample size was determined according to a G-power analysis (estimated effect size of *d* = 0.7, desired power effect of 0.80, and alpha level of 0.05). The participants were required to have engaged in exercise in the past and to exhibit healthy cardiopulmonary functioning. No data were excluded from analyses in “Study 1.”

#### Measures

Four exercise-related images were displayed using Samsung computers (screen: 22 inches with a 1,920 × 1,080-pixel resolution) to prime the participants to engage in mental simulation.

#### Procedures

The participants were randomly assigned to either the primed group or the control group and were shown the same four images; see “Appendix Presentation 1” for research program details. Then, all the participants indicated their intentions to engage in exercise-related behavior based on 10 items (e.g., “I now want to engage in exercise…” 1 = *not at all*, 9 = *very much*). The average score of the 10 items (Cronbach’s α = 0.80) was used as the dependent measure, with higher values denoting stronger intentions to exercise. An additional item (“Finally, how vivid was your mental imagery experience.”1 = *not very vivid*, 9 = *very vivid*) was used to quantify how vividly the participants simulated outcomes (*M* = 6.50, *SD* = 1.54) but was removed for the control group. Finally, the participants were debriefed, compensated, and dismissed.

#### Data Analysis

Text written by the experimental group was screened for keywords such as “sports,” “exercise,” “running,” “gym,” and “jogging,” while that of the control group was screened for keywords such as “light,” “dark,” “clear,” and “bright.” No data were rejected.

All statistical analyses were conducted with SPSS (version 25.0). We performed a descriptive statistics analysis and independent-sample *t*-tests for the priming effect of mental simulation.

#### Results

The results shown in Table 1 demonstrate that mental simulation priming (*M* ± *SD* = 6.11 ± 1.25) had a stronger effect than the control treatment (*M* ± *SD* = 4.90 ± 1.30, *p* < 0.05) on intention to exercise. Thus, we found a positive priming effect of mental simulation on intentions to exercise, verifying the assumptions of “Study 1.”

**TABLE 1:** *T*-tests on mental simulation priming on the intention to exercise.

	*M* ± *SD*	*n*	*t*	*p*
Controlled	4.90 ± 1.30	36	−4.02**	0.00
Primed M.S.	6.11 ± 1.25	36		

*p < 0.05; **p < 0.01; ***p < 0.001, the same as below.

#### Discussion

In “Study 1,” mental simulation mainly involved process simulation, which essentially allows one to imagine the process involved in reaching a set goal (Pham and Taylor, 1999). After rehearsing actions in their minds, individuals are more likely to confidently execute such actions. Compared with those not imagining certain actions in advance, those performing mental simulations beforehand can predict and plan for future events, enhancing their self-confidence and self-efficacy in engaging in healthy behaviors. A study of skills of doctors found that mental simulation plays three important roles for medical staff: providing feedback, enhancing insight, and increasing self-confidence (Schwindt and Mcnelis, 2015). Another study (Louridas et al., 2015) had surgeons use mental imagery to practice surgical skills in crisis situations. Surgeons using mental imagery exhibited stronger surgical skills in critical situations, while surgeons with regular training did not show an improvement and performed worse in crisis situations.

Mental simulation relies on complex experiences of bodily sensations and cognition, which can include physical sensations, feelings, and imagined scenarios. In other words, it is embodied (Barsalou, 2008). Given that mental simulation has a priming effect on intention to engage in exercise, which form of mental simulation is most effective at promoting such intention?

## Study 2

Semantic and image priming have been applied (de Koning et al., 2016; Mannaert et al., 2017; Wilson-Mendenhall et al., 2018) to improve exercise intentions and behaviors, but video priming has rarely been compared with the other methods. We applied a one-factor between-subjects experimental design with mental simulation priming methods (semantic, image, and video priming) used as the independent variable and intention to engage in exercise used as the dependent variable in “Study 2.” While “Study 1” explored forms of mental simulation most effective at influencing intentions to engage in exercise, “Study 2” explored the most effective priming approaches. We assumed video-primed mental simulation to have the strongest effect on increasing intention to exercise, followed by image- and semantically primed mental simulation.

### Methods

#### Participants

The study sample initially included 100 college students from a university in Beijing and a college in Shaanxi Province; some voluntarily participated, while others were given course credit for their participation. The other requirements were the same as in “Study 1.” Only one participant was excluded due to incomplete data, leaving 99 participants (77 female participants, *M*_age_ = 20.10, and *SD* = 2.18) in our final sample. This sample size allowed us to detect large effects (*f* = 0.40) at a 95% power level and medium effect sizes (*f* = 0.25) at a power level of 58%.

#### Measures

##### Exercise imagery ability scale

The exercise imagery ability scale compiled from the sport imagery questionnaire (SIAQ; Williams and Cumming, 2011) was used to more accurately measure imagery ability relative to the exercise scenes (“Appendix Data Sheet 1). There are 15 items (e.g., “Developing plans/strategies in my head.” 1 = *hard to image*, 7 = *easy to image*) on the scale. The first common factor explained 49.95% of the variance across items (Hair et al., 1998), and the internal consistency of the whole scale was alpha = 0.93.

#### Procedures

The participants in the semantic group read content describing an exercise experience. In the video-based group, the participants watched a 60 s video of the above-described process. The video was published online, and a gym workout and outdoor jogging were recorded. The participants were asked to simulate these scenarios as a single experience. More details about the procedures can be found in the “Appendix Presentation 2.” The participants finally completed the exercise-related questionnaire (e.g., “I now want to engage in exercise …” 1 = *not at all*, 9 = *very much*, Cronbach’s alpha = 0.86). The participants were debriefed, compensated, and dismissed.

#### Data Analysis

Text written by the participants was screened for keywords related to “sports,” “exercise,” “running,” “playing basketball,” etc. Data not pertaining to these keywords were removed. One participant in the image group was excluded from the study because of missing data, leaving data for a sample of 99 participants. One-way ANOVA was conducted with SPSS (Version 25.0).

#### Results

##### Manipulation checks

Levels of imagery ability reported by the groups did not show significant differences [*F*(2, 96) = 0.54, *p* = 0.59 > 0.05; *M*_*image*_ = 4.84, *SD* = 0.89, *M*_*semantic*_ = 4.70, *SD* = 1.24, *M*_*video*_ = 4.98, *SD* = 1.16], which were expected and showed that the participants had strong imaginations.

##### Descriptive statistics and one-way ANOVA

The results (Table 2) showed that the video-primed condition increased intention to exercise (*M* = 6.62, *SD* = 1.09) more than the image- (*M* = 5.66, *SD* = 1.54) and text-primed conditions [*M* = 5.40, *SD* = 1.60, *F*(1, 98) = 6.77, *p* < 0.01].

**TABLE 2:** One-way ANOVA: the influence of mental simulation priming modes on intentions to exercise.

Measure	Exercise intention		*F* (2.96)
	*M*	*SD*	
Priming mode			6.77**
Image	5.66	1.54	
Semantic	5.40	1.60	
Video	2.65	1.09	

The exercise intention was only measured once. Time window was too short before and after the manipulation, also random assignment assumes the groups were comparable. **p < 0.01.

A further analysis (Table 3) showed that video priming increased intention to exercise significantly more than mental simulation *via* image (*MD* = 0.97, SE = 0.35, *p* < 0.05) and text priming (*MD* = 1.23, SE = 0.35, *p* < 0.01), while differences observed between the text and image conditions were not significant (*p* > 0.05).

**TABLE 3:** Multiple comparisons of one-way ANOVA.

Prime mode		Exercise intention		*p*
		*M.D.*	*S.E.*	
Image	Semantic	0.26	0.35	0.46
	Video	−0.97*		0.01
Semantic	Image	–0.26		0.46
	Video	−1.23*		0.00
Video	Image	0.97*		0.01
	Semantic	1.23*		0.00

*p < 0.05.

#### Discussion

The results of “Study 2” showed that video-primed (*MD* = 1.23, SE = 0.35, *p* < 0.01) mental simulation most increased intention to exercise, supporting the experimental hypothesis. Early studies showed that, when reading text, individuals can use mental simulation to facilitate their comprehension. However, individuals experience different levels of spatial mental simulation during semantic content exposure (Liu and Bergen, 2016), leading to misunderstanding or ambiguity. On the one hand, the features of sentences themselves (e.g., grammar, the use of figurative or abstract words, and object sizes) (de Koning et al., 2016) have a moderating effect on the specific effects of mental simulation. On the other hand, differences between participants themselves also affect mental simulation, such as sensitivity of an individual to language, sense of space and time, past personal experiences, and social culture. When reading sentences, individuals do not merely simulate what is described; hidden content not explicitly referenced can also be perceived for mental simulation to refine and enrich their understanding (de Koning et al., 2016).

In images, as visually rich stimuli, specific visual information is directly presented. Individuals can then capture key features more quickly and store, process, and extract them from the brain. Feiereisen et al. (2008) studied the relationship between ability of consumers to understand new products and mental simulations based on differences between semantic content (sentences) and images. The results indicate that individuals spend less time processing information from images than they spend processing information from text. When individuals are learning a new task, they can understand and imitate this task based on text or images. When presented with text first, when the learned action is different, individuals actively modify their recollection of the text, and when they observe an action-focused image first, when the action learned is different, individuals are less proactive in correcting their memory of the image (Ianí et al., 2019). Therefore, images are more stable primers than text during exercise-related action mental simulation, controlling differences in individual simulation.

In terms of dynamic and continuous video simulation, when the content of mental simulation is vividly presented to individuals, mental simulation is primed faster. Allowing individuals to more directly and quickly enter a lifelike mental simulation saves time for semantic understanding, the dynamic transformation of images, the connection and transfer of plots, and the use of content for mental simulation. A study of mental practices used by gymnasts to learn new movements compared video simulations with ordinary exercises and found mental effects of the former to be superior (Battaglia et al., 2014) and more accurate in terms of tactile, visual, auditory, spatial, and time features. These features afforded the participants more realistic and accurate mental simulations. The results of “Study 2” also confirm that video-primed mental simulation better promotes intention to exercise than image- and semantically primed mental simulations.

“Study 2,” in verifying that mental simulation has a priming effect on intention to engage in exercise, shows video stimuli to have a stronger priming effect than image or semantic stimuli. Video priming was, therefore, chosen as the main method for the remainder of the research program.

## Study 3

Specific mental simulation skills may improve the cognitively based elicitation of exercise intention and behavior. The study was conducted using a 2 (process simulation: smooth vs. challenging) × 2 (outcome simulation: positive vs. negative) between-subjects design focused on the effects of different forms of mental simulation on exercise intention and exercise behavior. We hypothesized that mental simulation based on positive outcomes following challenging processes would promote intentions to exercise, while mental simulation based on negative outcomes following *smooth* processes would be the second most effective.

### Methods

#### Participants

Following the G × Power analysis (estimated effect size of *f* = 0.25, desired power effect of 0.85, and alpha level of 0.05), 155 students (130 female participants and *M*_age_ = 19.82 ± 1.97) were recruited from a University in Beijing and a University in Shaanxi; three were excluded due to incomplete data. The participants had past experience with exercise and were in good cardiorespiratory health.

#### Measures

Videos were updated from social media platforms so that the simulation materials would cover process and outcome simulations. All the videos included the same background music and lasted approximately 120 s.

##### The positive and negative affect schedule

To investigate how positive emotion moderates mental simulation impacts intention to exercise, we applied the PANAS (Watson et al., 1988) to measure the emotional differences between the participants.

#### Procedures

After reading and signing an informed consent form, the participants were randomly assigned to four groups and reported their emotions in PANAS as a baseline. In this study, the alpha coefficients were 0.89 and 0.92 for the positive and negative affect subscales, respectively.

The participants were shown the corresponding priming video for mental simulation and imagined themselves engaging in integrated exercise as vividly as possible. For more research program details, please see (“Appendix Presentation 3”). They then indicated their intentions to exercise based on 10 items (Cronbach’s alpha = 0.88). Our dependent variable was the average value of these 10 items, with lower values indicating stronger intentions to exercise. Finally, the participants reported on their emotional state by filling out the PANAS and were then debriefed, compensated, and dismissed.

#### Data Analysis

Analysis of covariance on the basis of descriptive statistics was conducted to test the effect of different types of mental simulations on exercise behavior after controlling the pretest emotions. All analyses were conducted with SPSS (Version 25.0). For further analysis, we controlled for pretest positive emotions as a covariable to explore the role of posttest positive emotions in imagery ability and intention to exercise. Data analysis was conducted using Process 3.3 (Hayes, 2013) for SPSS.

#### Results

##### Imagery ability manipulation

The participants reported on their imagery ability (*M* = 4.84 ± 0.94) and intentions to exercise (*M* ± *SD* = 5.93 ± 1.36).

##### Mental simulation effect on exercise intention irrespective of posttest positive emotions

Posttest positive emotions were used as a covariable. The participants who simulated the *smooth* process with a negative outcome (*M* = 6.22 ± 0.20, 95% *CI*: 5.83–6.61) showed stronger intentions to exercise than those who imagined a *smooth* process with a positive outcome (*M* = 5.72 ± 0.21, 95% *CI*: 5.31–6.14). The participants who simulated challenging exercise with a positive outcome (*M* = 6.05 ± 0.20, 95% *CI*: 5.65–6.44) showed stronger intentions to exercise than those who simulated challenging exercise with a negative outcome (*M* = 5.64 ± 0.22, 95% *CI*: 5.21–6.06). This outcome indicates an interaction effect between process and outcome simulation [*F*(1,151) = 4.83, *MS* = 7.64, *t* = −2.20, *p* = 0.03 < 0.05, 95% *CI*: -1.72, -0.91, η*_*p*_*^2^ = 0.03]. We found no main effects of process [*F*(1,151) = 0.05, *MS* = 0.07, *t* = 1.39, *p* = 0.83 > 0.05, 95% *CI*: -0.17, 0.99, η*_*p*_*^2^ = 0.00] or outcome simulation [*F*(1,151) = 0.40, *MS* = 0.63, *t* = 1.39, *p* = 0.53 > 0.05, 95% *CI*: -0.28, 0.54, η*_*p*_*^2^ = 0.00]. Positive emotions had a significant impact on behavioral intentions [*F*(1,151) = 25.58, *MS* = 40.48, *t* = 5.06, *p* < 0.00, 95% *CI*:0.05, 0.12, η*_*p*_*^2^ = 0.15].

##### Positive emotions had a mediating effect between imagery ability and exercise intentions

Prepositive emotion: the results showed a significant mediating effect (*effect* = 0.17, *Bootstrap SE* = 0.06, 95% *CI*: 0.07, 0.30) of posttest positive emotions between imagery ability and intention to exercise (Table 4). At the same time, imagery ability had a significant direct effect (*effect* = 0.55, *SE* = 0.12, *t* = 4.43, *p* < 0.001, 95% *CI*: 0.30, 0.79) on the intention to exercise, showing a partial mediating effect of positive emotions between imagery ability and the intention to exercise after controlling for the prepositive emotion. The mediating model showed a good degree of fit [*R*^2^ = 0.29, *MSE* = 1.35, *F*(3,147) = 19.65, *p* < 0.001].

**TABLE 4:** Mediation analysis: mediating effect of positive emotions between imagery ability and exercise intentions.

Effects	Estimate	*SE*	95% CI	
			*LL*	*UL*
Total effect	0.72	0.12	0.49	0.95
Direct effect	0.55	0.12	0.30	0.79
Indirect effect	0.17	0.06	0.07	0.30

*Total N = 151, one participant missed the emotion data was excluded. CI, confidence interval; LL, lower limit; UL, upper limit.*

#### Discussion

**FIGURE 1:**
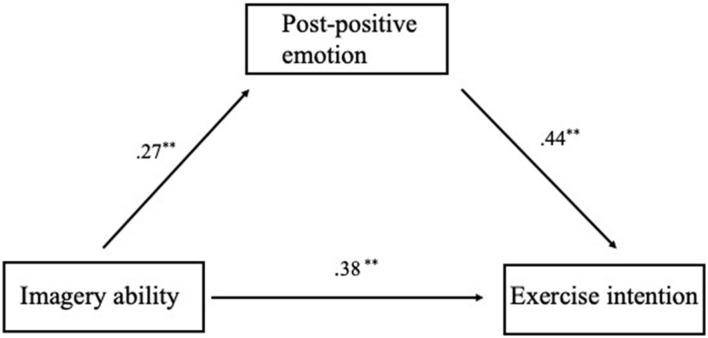
A mediation model of positive emotion between imagery ability and exercise intention. Pre-positive emotion is controlled for in the regression of post-positive emotion (coefficients = −0.35, *p* < 0.01) and exercise intension (coefficients = −0.72, *p* < 0.001).

The results of “Study 3” showed that process- and outcome-based simulation had a salient interaction effect on the intention to exercise rather than main effects. Of the four types of mental simulation tested, the *smooth* process with a negative outcome and the challenging process with a positive outcome increased exercise intention to the same degree. Individuals generally agree on the importance of healthy behaviors such as exercising and eating healthy food but still struggle with eating junk food, working out in gyms, and limiting their alcohol intake (Reynolds et al., 2017). The extra mediation model (Figure 1) showed that imagining positive behaviors leads to positive emotions surrounding exercise (Wilson et al., 2017). Additionally, when individuals experience positive effects rather than fluctuating effects, they are more likely to engage in daily exercise (Sweeney and Freitas, 2018). These studies corroborate our model in showing that imagining exercising induces positive emotions, which indirectly increases intention to exercise. In the present study, we did not test the interaction of positive emotions and imagery ability on exercise intentions. Since it was not our mainly focus, further study could put an attention on this.

“Study 3” did not fully validate our experimental hypothesis. Previous studies have shown that, when simulating future events in daily life, simulating challenging experiences with positive results better prepares one for future events (Jeannerod, 2001). This phenomenon demonstrates that the challenging process of simulating an event in advance can provide individuals with alternative solutions for, when the actual event occurs through the problem-solving functions of mental simulation, preventing the occurrence of negative outcomes. At the same time, a positive simulation of a pleasant experience and positive emotions (Hagger, 2017) can motivate individuals to achieve their goals.

## Study 4

In a study applying mental simulation to encourage exercise behavior, we explored the effects of mental simulation interventions (a *smooth* process with a negative outcome and a challenging process with a positive outcome) applied over 1 week. We used a mixed experimental design with an independent variable of 2 (groups of mental simulation: a *smooth* process with a negative outcome, a challenging process with a positive outcome) × 2 (booster: one time, daily) × 2 (time: before and after intervention) and a dependent variable (an exercise score for 1 week) as measured by the International Physical Activity Questionnaire Short Form (IPAQ-SF; Rangul et al., 2008).

### Methods

#### Participants

**FIGURE 2:**
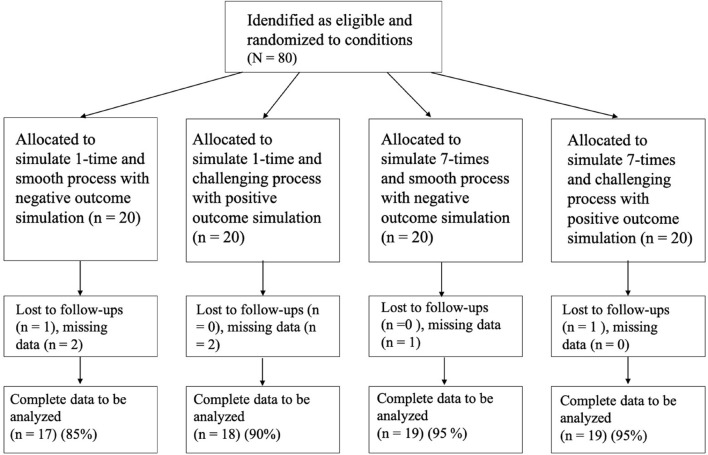
The process of recruiting participants of Study 4.

We recruited 80 students from a University in Beijing (Figure 2) with no cardiopulmonary disease and with exercise experience (G-power analysis estimated effect size of *f* = 0.25, desired power effect of 0.85, and an alpha level of 0.05). Five participants were removed from the study due to not reporting their levels of exercise after 1 week; another two participants reported a value of “0” before and after the experiment. After these omissions, data for 73 participants remained. The sample included 15 male students (20.5%) and 58 female students (79.5%). The participants were 21.09 ± 2.97 years of age on average.

#### Measures

The same experimental equipment employed in Study 1 was used. We used the short version of the IPAQ-SF (for the scoring algorithm; Fan et al., 2014; e.g., ‘‘How much time did you usually spend on one of those days doing vigorous physical activity as part of your work?’’ *____hours per day and ____* = *minutes per day.*) and otherwise used the same experimental materials employed in ‘‘Study 3.’’ The questionnaires were collected online. The number of WeChat^1^ steps was used as reference data for activity levels. One week of continuous mental simulation was administered using the “Lil’ Punch Card” function of WeChat.

#### Procedures

**FIGURE 3:**
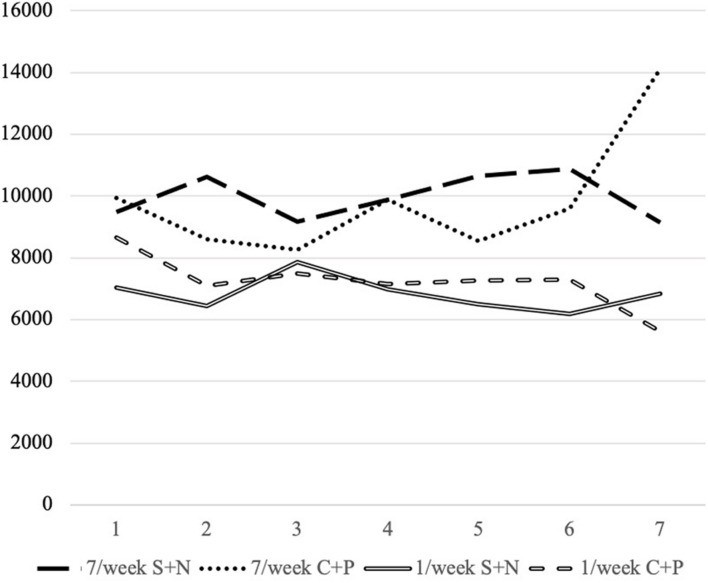
Steps taken weekly *via* mental simulation intervention.

The participants provided their informed consent. The IPAQ-SF was first measured as the baseline level. We then randomly divided the participants into four experimental groups. The participants watched a 120-s video while engaging in mental simulation and then recorded their thoughts as a journal entry, which they then sent to the experimenter. They were asked to check in on time on each day of the experiment and then simulated one time a day for 1 week. Their daily activity was reported to the facilitator with the number of WeChat steps taken as an indicator. Instances involving moderate- or high-intensity exercise (based on the IPAQ-SF) were reported to the experimenter. This scenario was not the case for the participants of the one-time simulation. The IPAQ-SF was then taken after 1 week. See Figure 3.

#### Data Analysis

All analyses were conducted with SPSS 25.0, and a repeated measures analysis of variance was performed based on descriptive statistics (Table 5).

**TABLE 5:** Descriptive statistics of exercise amount.

Booster	Group	Baseline P.A.			Posttest P.A.	
		*n*	*M*	*SD*	*M*	*SD*
Once	S + N	17	1,315.22	915.06	1,623.09	1,708.78
	C + P	18	1,515.92	1,122.71	1,572.89	1,488.79
Daily	S + N	19	1,452.32	1,103.69	2,617.82	1,708.78
	C + P	19	2,017.24	1,120.16	2,490.47	1,734.53

*Booster for times of mental simulation measurements. S, smooth process; C, challenging process; P, positive outcome; N, negative outcome.*

#### Results

The statistical results (Table 6) showed a main effect [*F*(1, 72) = 7.01, *p* < 0.05, η*_*p*_*^2^ = 0.09] of time for the group, but no other interaction effects. The main effect of the booster carried out between groups was found to be significant [*F*(1, 72) = 6.09, *p* < 0.05, η*_*p*_*^2^ = 0.08], the main effect of simulation groups was not found to be significant (*p* > 0.05), and no other interaction effects were observed. The participants completing more than one mental simulation intervention showed the most intense exercise behavior (*p* < 0.05), which was in line with our hypothesis. In terms of the groups of mental simulation, the results affirmed those of Study 3; rather, the booster executed has a significantly positive effect on levels of exercise, and mental simulations with this effect include those involving the *smooth* process with a negative outcome and those involving the challenging process with a positive outcome. We found no significant differences in means of exercise promotion.

**TABLE 6:** Repeated-measures ANOVA of exercise for different types and periods of mental simulation.

Variable	Source of variation	*SS*	*MS*	*df*	*F*	*p*	η_*p*_^2^
Within-subjects	Time	9138341.00	9138341.00	1	7.01*	0.01	0.09
	Time × Booster	2025005.87	2025005.87	1	1.55	0.22	0.02
	Time × Booster	3694202.86	3694202.86	1	2.83	0.10	0.04
	Time × group × Booster	443454.95	443454.95	1	0.34	0.56	0.01
	Error term	89969278.9	1303902.59	69			
Between-subjects	Group	787272.20	787272.20	1	0.32	0.57	0.01
	Booster	14810964.5	14810964.5	1	6.09*	0.02	0.08
	Group × Booster	187610.79	187610.79	1	0.08	0.78	0.00
	Error term	167792992	2431782.50	69			

Booster for times of mental simulation measurements. Booster for once a week mental simulation or daily a week mental simulation. Group for combination of different mental simulation. *p < 0.05.

The results based on the number of steps taken per week (Figure 3) showed that the one-time mental simulation intervention generally showed a declining trend. The participants engaging in mental simulation one time a day took more steps daily on average than the intervention group performing only one mental simulation, showing a stable trend overall. Mental simulation based on a positive outcome following a challenging process displayed a steep ascent on the last day, while mental simulation based on a negative outcome following a *smooth* process showed a descending trend. Comparing these trends with the average number of steps, we propose that a challenging process with a positive outcome mainly promotes adherence to exercise and that a *smooth* process with a negative outcome tends to increase the amount of exercise.

#### Discussion

The participants performing mental exercise simulation one time a day showed higher levels of exercise than those doing so only on the first day, which corroborated the findings of a previous study (Schendan and Giorgio, 2012). The within-factor booster did not show a significant effect, and time did prompt us that, to avoid the natural time effect, intervention time needs a proper extension. We found no differences between the *smooth* process with a negative outcome and the challenging process with a positive outcome, as in “Study 3.” The mental simulation of the *smooth* process with a negative outcome had a better effect on promoting exercise over the week-long intervention. This result is consistent with findings of Koka (2016) on engagement in muscle-strengthening exercise among adolescents. Mental simulation affects only strengthening exercises, and the frequency of exercise does not increase according to subsequent tracking. First, the intervention effect of mental simulation is short-lived and cannot be effectively maintained over a week. Second, repeated mental simulations can effectively maintain increased levels of exercise (Szpunar and Schacter, 2013). Finally, during simulation, imagining a *smooth* process can improve one’s sense of competence in performing exercise, promoting a superior and more complete exercise plan. In addition, after simulating negative results, individuals actively avoid engaging in behaviors that will lead to negative outcomes and constantly remind themselves to adopt goal-oriented behaviors and monitor their habits to achieve a healthy lifestyle.

## General Discussion

Mental simulation is often involved in decision-making (Steinmetz et al., 2018), consumption (Castaño et al., 2008), skill learning (Louridas et al., 2015), eating habits (Petit et al., 2016), language comprehension, artificial intelligence (Langley et al., 2016), etc. The detailed simulation process leads to a stronger intention for people to transfer into behaviors. As expected, video-primed simulation has the best priming effect among semantic-, image- and video-primed methods. Positive emotion plays a mediating role between mental simulation and exercise intentions, which was the same as we assumed.

The role of mental simulation in promoting engagement and intentions to engage in certain behaviors in the future has been confirmed by many previous studies (Pham and Taylor, 1999; Castaño et al., 2008; Stragà and Ferrante, 2014; Koka, 2016; Koka and Hagger, 2016; Petit et al., 2016). One exception to this was a study by Meslot et al. (2016), which reported null results. Our study went beyond by testing different types of simulations to promote physical activity, since process and outcome simulations have different effect on intentions (Pham and Taylor, 1999). If the purpose of the research is to promote physical activity, then it makes sense to choose a more suitable type of simulation for research. In this study, the participants not only paid attention to themselves but also to other factors that might affect themselves—“visualization” paths, based on imagery-based techniques such as mental contrasting, are more conducive to strengthening motivation and promoting exercise intentions. It is faster and easier for a person to imagine in more detail preparing for a goal and simulate the situation of whether or not the goal is achieved, and video-primed material shows a more promoting effect than semantic- and image-primed material. However, whether the impacts are positive or negative depends on the nature of a given event. For example, an individual may simulate the visceral experience of eating a favorite food based on its taste, smell, and texture. Such simulation will lessen his or her desire for this food and decrease his or her intake (Muñoz-Vilches et al., 2019). The mental simulation of goals that do not require long-term effort or physical or mental labor usually reduces the actual desire for them (Muñoz-Vilches et al., 2019). However, mental simulation may have a positive effect on encouraging individuals to realize goals requiring planning and sustained effort (Pham and Taylor, 1999).

Developing sustainable exercise takes a certain degree of persistence. The promotion or maintenance of behaviors by mental simulation has been explored in previous studies (Pham and Taylor, 1999; Louridas et al., 2015; Hammell and Chan, 2016; Koka and Hagger, 2016). Unlike past studies, the present work focused on exercise and confirmed the effectiveness of using mental simulation to promote exercise.

The self-model reflects one’s feelings regarding whether one is worthy of love and respect, which are related to past experiences (Yang et al., 2013). Mental simulation affects not only the experience of exercise but also the development of goals to exercise more. According to our simulation results, a positive body-image model encourages individuals to approach their goals, while a negative model makes individuals more likely to adopt avoidance strategies. Mental simulation based on process simulation, as an alternative mental schema, is also referred to as indirect experience, which is one’s internal and indirect observations of behaviors of others and of one’s own abilities. In our *smooth* process simulation, our participants fully previewed the psychological and physical states and social environments required to exercise, and positive emotional states enhanced their sense of self-efficacy in addressing expected future events (Pham and Taylor, 1999).

The theory of planned behavior asserts that behaviors are directly controlled by intention with sufficient actual controlled conditions, such as personal capabilities, opportunities, and resources. A more positive attitude, more support from others, and stronger perceived behavior control lead to more intense behavior intention. Various external and uncertain factors affect the development of an event. For example, people exercising outdoors in the summer cannot fully predict whether it will rain, as frequent and irregular rainfall may be typical in one’s environment. Should we, therefore, abandon planned outdoor activities? Challenging process simulations can allow individuals to practice adjusting their physical and mental states to make goal-oriented behavior choices as unexpected events arise rather than giving up.

While it may be effective for routine actions to simulate only successful actions, in the case of non-routine, less predictable sports, it would be of great value to develop strategies for managing unexpected events in advance. For most individuals, processes involved from preparing for exercise to maintaining an exercise regime cannot be accurately predicted. Even intentions to exercise can fluctuate, making individuals more apt to use the results of mental simulation to set goals for themselves.

## Limitations

This study has some limitations. First, it conducted a short-term intervention due to the unexpected COVID-19 epidemic. Further study focusing on the long-term intervening influence of mental simulation on exercise using an intervention of more than 3 months is suggested (Helgadóttir et al., 2018).

Second, the samples selected in the present study were mainly college students, which mainly focused on a specific group. Also, the number of samples in “Study 1” needs to be increased to make results more reliable. Further study should cover a variety of participants to achieve a comprehensive analysis of the effect that mental simulation promotes exercise intention and behavior.

Third, in tracking exercise behavior, there may have been deviations in the self-reported scale. In addition, this study did not account for variations due to social or individual expectations leading to data inaccuracies. While having each participant wear an accelerometer would have been an effective means to track exercise behavior (Eisenberg et al., 2017), it would have been prohibitively expensive due to the number of participants involved in this study. In the future, it will be necessary to develop more economical and accurate standards and methods for measuring exercise behavior.

## Broader Implications and Avenues for Future Research

In this study, process simulations were divided into smooth and challenging processes to reflect actual conditions, and we further applied simulations with different outcomes (positive and negative). On this basis, we examined the simulation of exercise. Previous studies have mostly focused on action-based representations (e.g., movement imagination, skill development). Mental simulations in this study involved imagining not only exercising but also associated perceptions, sounds, images, emotions, etc. Subsequent studies may, therefore, build upon Studies 3 and 4 to test designs of further complexity.

Although “Study 4” controlled for baseline exercise frequency, stages of experience of participants with regular exercise also remain to be investigated. A previous study (Phillips et al., 2016) suggested that intrinsic motivation influences exercise frequency through intention for exercise initiators and through habit for maintainers. In the same fashion, mental imagery could not only influence adopters beyond intention but also influence maintainers differently, possibly through habit. This possibility deserves a deeper exploration.

## Conclusion

This study contributes more evidence to current knowledge of the unique effects of mental simulation on intention to exercise. The present work ultimately divided process mental simulation into simulations of *smooth* and challenging processes and forms of outcome mental simulation into simulations of positive and negative outcomes. Not only does it offer another confirmation that exercise intentions could be increased with mental simulation, but it also encourages to use video-priming for stronger effects instead of semantic or image-priming. It also underlines that mental simulation interventions are most effective for exercise when practiced multiple days in a row instead of one time a week. Most importantly, it highlights that mental simulation interventions will work best to promote exercise adherence if they balance process and outcome components, like combining a challenging process with a positive outcome, or a smooth process with a negative outcome. In other words, envisioning that a difficult experience can end positively, or that an easy experience can still end negatively, is more helpful than unconditionally optimistic or tough scenarios.

## Author’s Note

All images in our article were downloaded from the open source website (https://v.youku.com/v_show/id_XMzcwNDU0MDQ2NA==.html). Some of the images were downloaded with public authority.

## Data Availability Statement

The original contributions presented in the study are included in the article/Supplementary Material, further inquiries can be directed to the corresponding author.

## Ethics Statement

The studies involving human participants were reviewed and approved by the Ethics Committee of the School of Psychology at the Beijing Sport University. The patients/participants provided their written informed consent to participate in this study.

## Author Contributions

WZ conducted the experiments, collected and analyzed the data, carried out the bulk of the literature review, and wrote the manuscript. GZ played an editorial role in writing – review and editing and participated in checking methods and guiding WZ during the data analysis. Supervised the present study. WZ and GZ designed the current study, the methods of experiment, read and approved the final manuscript. Both authors contributed to the article and approved the submitted version.

## Conflict of Interest

The authors declare that the research was conducted in the absence of any commercial or financial relationships that could be construed as a potential conflict of interest.

## Publisher’s Note

All claims expressed in this article are solely those of the authors and do not necessarily represent those of their affiliated organizations, or those of the publisher, the editors and the reviewers. Any product that may be evaluated in this article, or claim that may be made by its manufacturer, is not guaranteed or endorsed by the publisher.

## Footnotes

1 WeChat is a social media with a high penetration rate in the Chinese-speaking circle, and the “WeChat sport” has a step counter feature.
