# Supplementary material for: Mental Simulation to Promote Exercise Intentions and Behaviors
Source: Front Psychol. 2021 Nov 16;12:589622. doi: 10.3389/fpsyg.2021.589622 (PMC8637839; doi:10.3389/fpsyg.2021.589622)
Supplement: Supplementary file 1 [file Data_Sheet_1.PDF]

Exercise Imagery Ability Scale

(Chinese version compiled from SIAQ; Williams & Cumming, 2011)

1.Making up plans/strategies in my head

2. Giving 100% effort even when things are not going well

3.Refining a particular exercise skill

4. The positive emotions I feel while doing my exercise

5. Achieved a staged success

6. Alternative plans/strategies

7. The anticipation and excitement associated with my exercise

8. Improving a particular skill

9. People around me gave me a lot of positive feedback

10. Remaining positive after a setback

11. The excitement associated with exercising

12. Making corrections to physical skills

13. Creating a new exercise plan

14. Achieve my exercise goal

15. Remaining confident during a challenging situation

7-Likert Scale, 1 = Hard to image, 7 = easy to image.
