# Supplementary figures and images for: Mental Simulation to Promote Exercise Intentions and Behaviors
Source: Front Psychol. 2021 Nov 16;12:589622. doi: 10.3389/fpsyg.2021.589622 (PMC8637839; doi:10.3389/fpsyg.2021.589622)

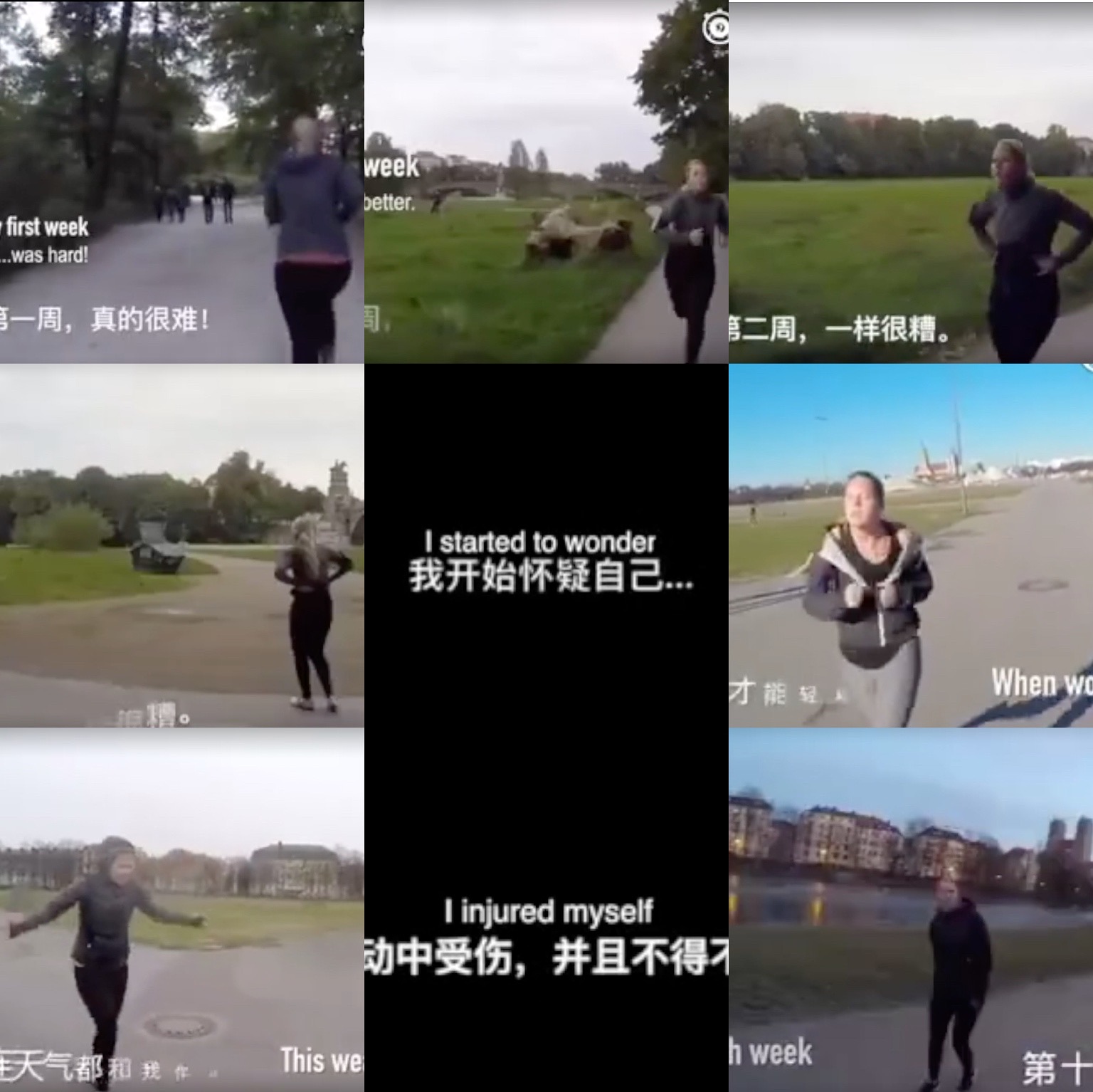

Supplement: Supplementary file 2 [file Data_Sheet_2.ZIP › materialsdata/challengingprocess.JPG]

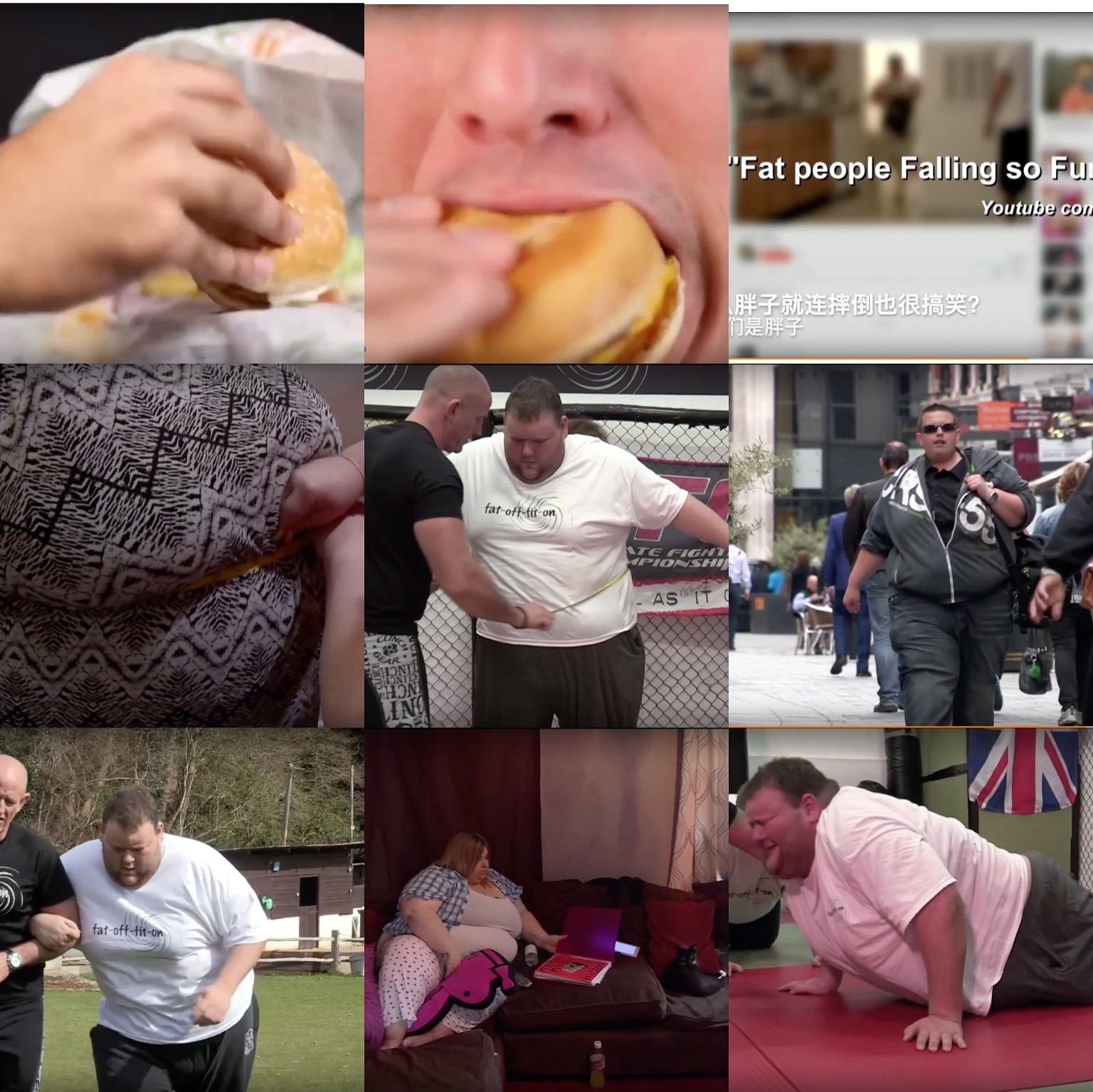

Supplement: Supplementary file 2 [file Data_Sheet_2.ZIP › materialsdata/negativeoutcome.JPG]

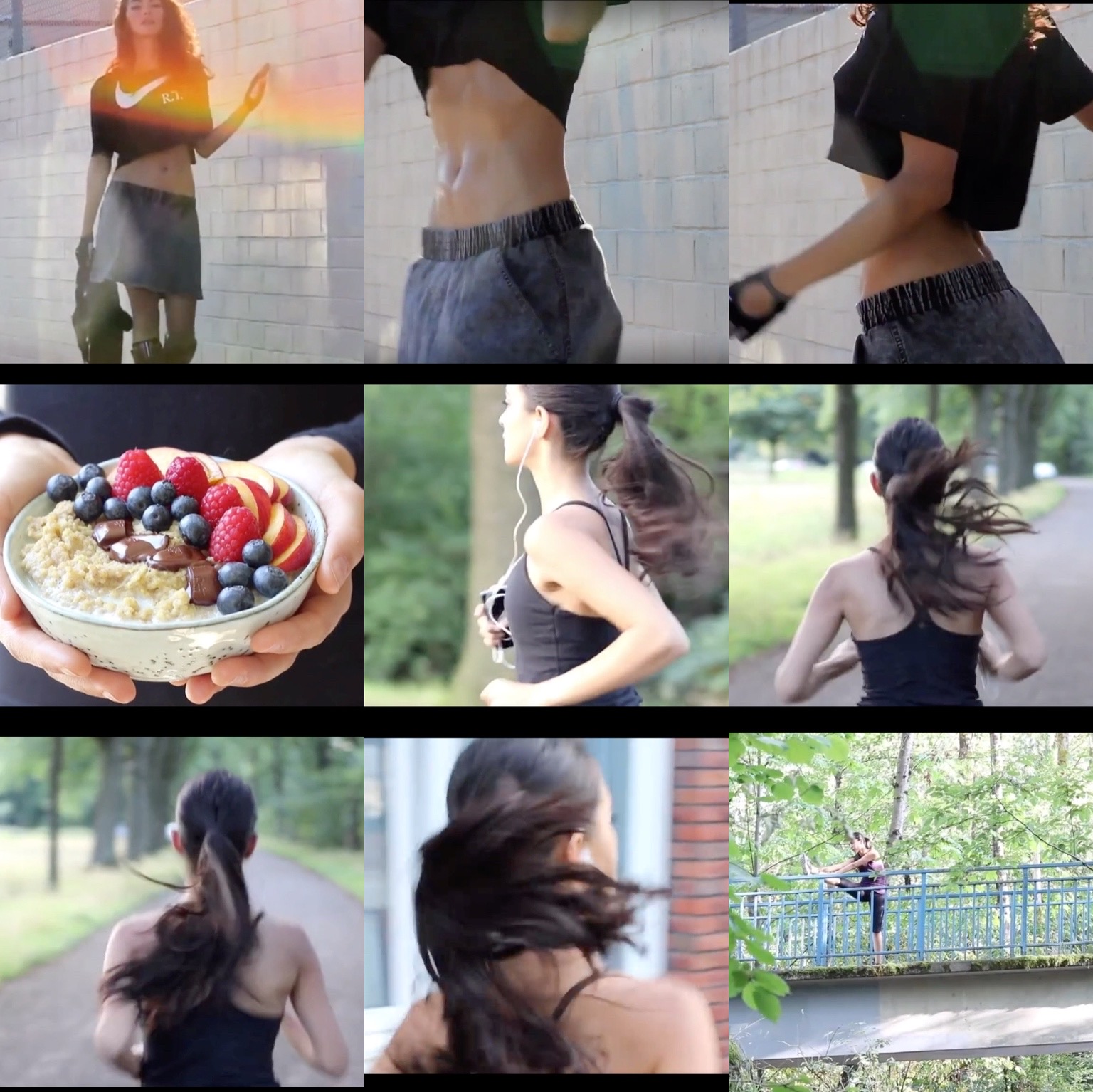

Supplement: Supplementary file 2 [file Data_Sheet_2.ZIP › materialsdata/positiveoutcome.JPG]

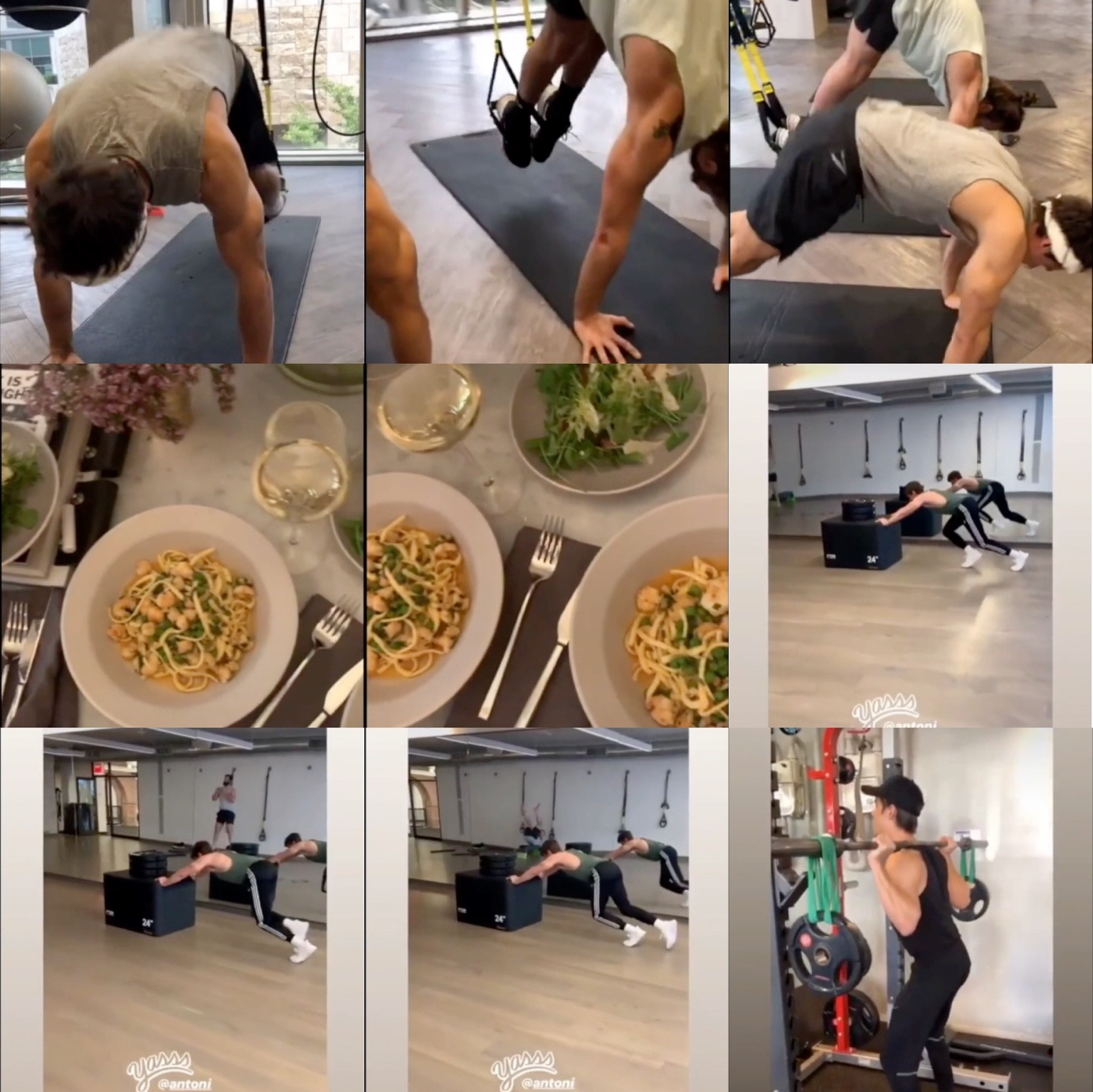

Supplement: Supplementary file 2 [file Data_Sheet_2.ZIP › materialsdata/Smoothprocess.JPG]
