# Supplementary material for: Mental Simulation to Promote Exercise Intentions and Behaviors
Source: Front Psychol. 2021 Nov 16;12:589622. doi: 10.3389/fpsyg.2021.589622 (PMC8637839; doi:10.3389/fpsyg.2021.589622)

(Original in Chinese)
BEIJING SPORT UNIVERSITY

Research Program of Study 3

I am a master student from school of psychology, Beijing Sport University. We are planning to conduct a research study, which I invite you to take part in. This form has important information about the study, what we will ask you to do if you decide to be in this study, and the way we would like to use information about you if you choose to be in the study.

Why are you doing this study?

You are being asked to participate in a research study about imagining exercise behavior.

What will I do if I choose to be in this study?

You will also be asked to imagine an exercise situation. After that, we will ask you a few questions about your personal choice in exercise intention.

Study time:

Study participation will take no more than 10-20 minutes.

What are the possible risks or discomforts?

The risks to your participation in this study are mild mental stress. The only benefit to you is that you get an experience of participating in the experiment, and the results of the experiment will be useful to people in the future.

How will you protect the information you collect about me, and how will that information be shared?

Results of this study may be used in publications and presentations. Your study data will be handled as confidentially as possible. If results of this study are published or presented, no individual names or other personally identifiable information will used.

Financial Information

Participation in this study will get remuneration.

What are my rights as a research participant?

Participation in this study is voluntary. If at any time and for any reason, you would prefer not to participate in this study, please feel free not to.

<u>Who can I contact if I have questions or concerns about this research study?</u>

If you have questions, you may contact the researcher Weitan Zhong at zwttt0501@hotmail.com

<u>Consent Section</u>

By accepting this HIT you are consenting to participation in this study.

Questionnaire for Study 3

Demographics

First, please answer a few questions about yourself.

What is your gender?

Age_____

Instruction

In next page you will see a watch a 120-second video. Your task is to imagine yourself in the environment described in the video, be sure to simulate an exercise scenario from the first-person perspective. In other words, imagine that you are in it and can see the surrounding environment through your own eyes. Imagine what you will experience if you are really in the scene, consider what you will do and how you will feel. These can be the exercises given in the material, or you can usually do it. Try to simulate the whole process of doing an exercise.

Please click when you are ready to begin.

You will have 120 seconds imagine your experience in this environment, and then completed the Exercise Imagery Ability Scale fist, write your thoughts down in the designated space (more than 100 words).

At the end of the survey, we will ask you a few questions about your experience.

1. Do you care about your weight?

○ Yes.

○ No (jump to 5).

2.Right now, you would like to lose weight

Not at all ○ ○ ○ ○ ○ ○ ○ ○ ○ Very much

3. Right now, you would like to maintain the current weight

Not at all ○ ○ ○ ○ ○ ○ ○ ○ ○ Very much

4. Right now, you would like to gain weight

Not at all ○ ○ ○ ○ ○ ○ ○ ○ ○ Very much

5. Do you pay attention to your physical exercise amount?

○ Yes.

○ No. (jump to 9)

6. Right now, you would like to increase the amount of exercise

Not at all ○ ○ ○ ○ ○ ○ ○ ○ ○ Very much

7. Right now, you would like to maintain the current amount of exercise

Not at all ○ ○ ○ ○ ○ ○ ○ ○ ○ Very much

8. Right now, you would like to reduce the amount of exercise

Not at all ○ ○ ○ ○ ○ ○ ○ ○ ○ Very much

9. Right now, you would like to choose

Junk food ○ ○ ○ ○ ○ ○ ○ ○ ○ Healthy food

10. Right now, you would like to choose

Sugary drinks ○ ○ ○ ○ ○ ○ ○ ○ ○ Black coffee/water

11. Right now, you have intention to pay attention to exercise-related information

Not at all ○ ○ ○ ○ ○ ○ ○ ○ ○ Very much

12.Right now, you have intention to invest money in exercise-related products

Not at all ○ ○ ○ ○ ○ ○ ○ ○ ○ Very much

13. Right now, you have intention to pay attention to community / school medical service information

Not at all ○ ○ ○ ○ ○ ○ ○ ○ ○ Very much

14 Right now, you have intention to learn about sport medicine

Not at all ○ ○ ○ ○ ○ ○ ○ ○ ○ Very much

15. Right now, you have intention to achieve long-term exercise habits

Not at all ○ ○ ○ ○ ○ ○ ○ ○ ○ Very much

16. Right now, you have intention to engage in ______ activities after the experiment, and carry into execution after the experiment. (Please feedback to the experimenter via WeChat after the study, thank you for your cooperation!)

Not at all ○ ○ ○ ○ ○ ○ ○ ○ ○ Very much

17.The exercise you will do is______.

End

Thank you very much for participating!

Video for smooth process with positive outcome type mental simulation group

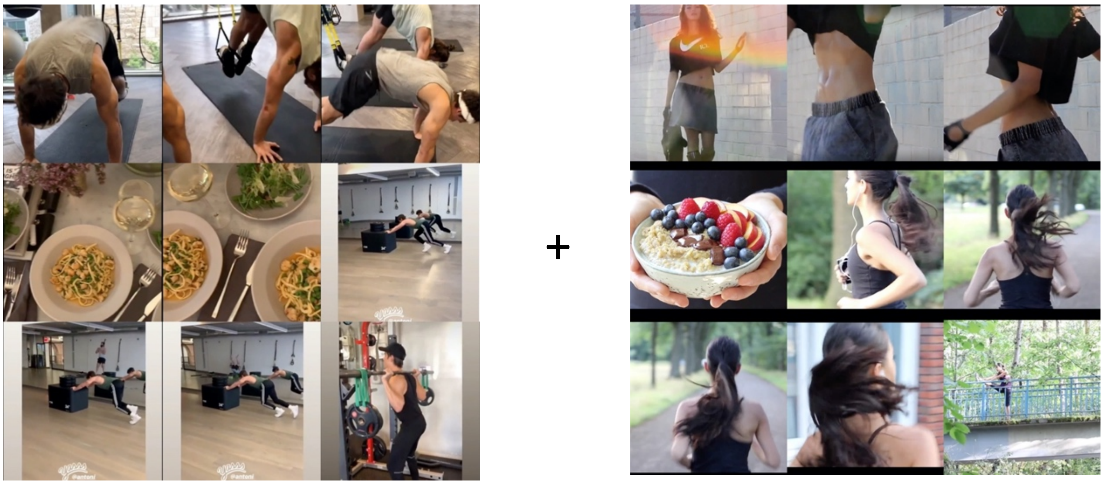

Video for smooth process with negative outcome type mental simulation group

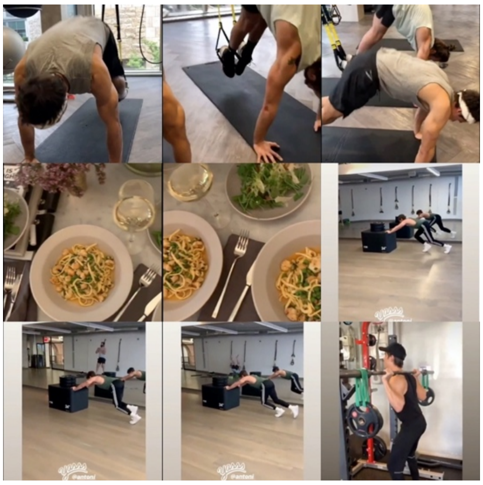

+

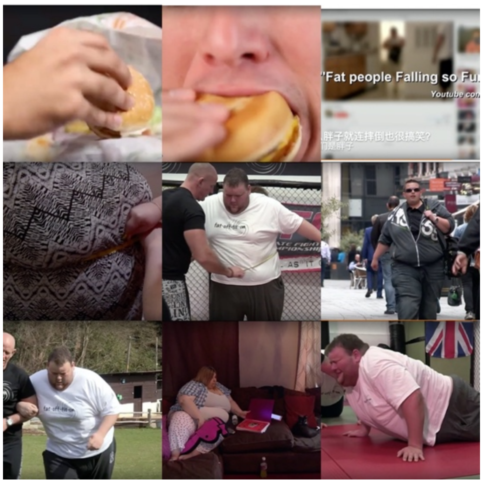

Video for challenging process with positive outcome type mental simulation group

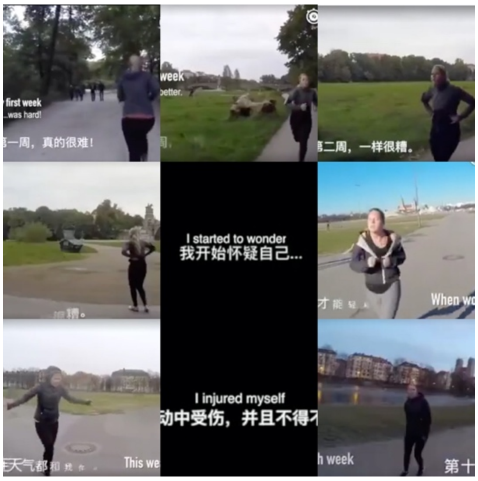

+

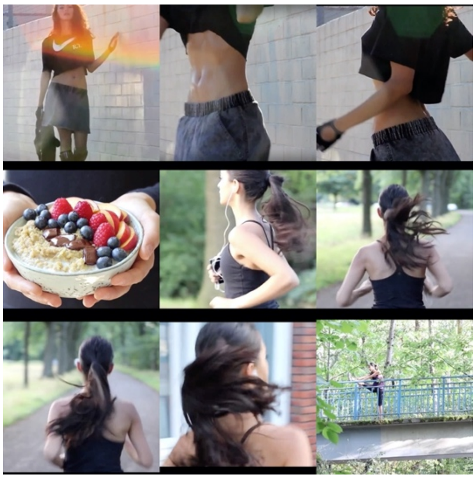

Video for challenging process with negative outcome type mental simulation group

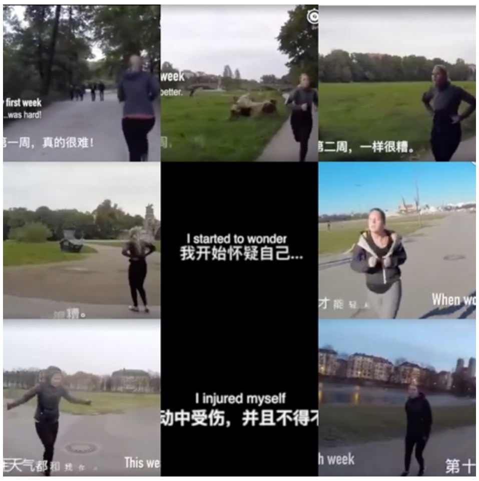

+

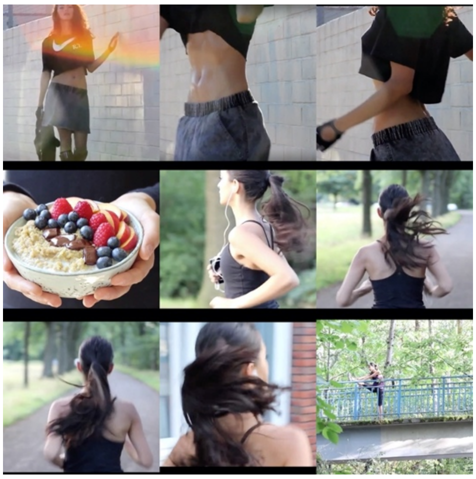

Supplement: Supplementary file 5 [file Presentation_3.PDF]
